# Stress and resilience among women living with HIV in Nigeria

**DOI:** 10.4102/phcfm.v11i1.2046

**Published:** 2019-10-23

**Authors:** Aliyu Adamu, Gugu Mchunu, Joanne R. Naidoo

**Affiliations:** 1 School of Nursing and Public Health, University of KwaZulu-Natal, Durban, South Africa; 2 Department of Nursing Science, Nelson Mandela University, Port Elizabeth, South Africa

**Keywords:** HIV, Nigeria, psychological resilience, stress, women

## Abstract

**Background:**

Psychological morbidities concurrent with HIV have been the focus of considerable scientific investigations. However, researchers have largely overlooked HIV-related stress and resilience among women living with HIV in rural communities.

**Aim:**

This study explored the associations between psychological resilience and HIV-related stress among women living with HIV.

**Setting:**

The study was conducted in three randomly selected hospitals that provide primary HIV care in Niger state, Nigeria.

**Methods:**

A predictive cross-sectional design was used to describe the relationship between perceived stress and resilience among the study population.

**Results:**

Out of 748 participants who completed the Connor–Davidson resilience scale and the perceived stress scale questionnaires, 676 returned the questionnaire in usable form. While the results showed moderate levels of perceived stress and a high level of psychological resilience, there was a significant and negative relationship between HIV-related stress and psychological resilience (*r* = -0.601, *p* = < 0.001). Also, higher resilience was significantly associated with decreased perceived stress.

**Conclusion:**

It is concluded that measures to promote resilience and employment opportunity may ameliorate HIV-related stress among women living with HIV.

## Introduction

Sub-Saharan Africa (SSA) remains the world’s most affected region by the HIV epidemic. It is reported that more than half of the 36.9 million people infected with HIV live in SSA.^1^ More than three decades after the discovery of HIV, sub-Saharan African women still account for over 56% of new cases of infection.^1^ The prevalence of the epidemic varies from one country to another in SSA. However, the prevalence of HIV and AIDS among women in the region calls for attention. Nigeria is ranked second with the highest number of cases globally after South Africa. The World Health Organization (WHO) progress report estimated the prevalence of HIV infection among Nigerian women to be 3.4% compared with a prevalence rate of 2.6% among men.^2^

The use of highly active antiretroviral therapy (HAART) has transformed HIV infection to a chronic disease.^3^ However, the chronicity is associated with emotional, psychological and physical challenges.^4^ Chronic health challenges are known to create emotional and psychological needs because of the burden of symptoms, treatment approaches and the effect of living with several diseases concurrently. Evidence has shown that the impact of managing a chronic disease is associated with mental health challenges, such as depression.^5^

High levels of stress are common among people living with HIV.^4^ However, in terms of gender, women experience more psychological stress.^6^ Stressors among women living with HIV (WLWH) may be HIV disease specific and non-HIV specific. HIV-specific stressors are directly related to HIV diagnosis and management, while non-HIV stressors are not directly related to HIV. The common stressors may include poverty, stigma, discrimination, violence and unemployment.^7^ Psychological stress among WLWH may be associated with the negative physical and psychosocial well-being of the individuals^7^ as well as the onset of psychiatric disorders.^5,7^ Qualitative evidence had shown that psychological stress among WLWH can compromise linkage and retention to HIV care, reduced health-related quality of life, increased risk of HIV transmission and depression.^7^

Although HIV infection is among the risk factors for chronic psychological stress among women in SSA,^7^ yet it is not all WLWH who experience chronic psychological stress or other mental health challenges associated with their HIV status. This highlights individual differences in response to trauma associated with HIV diagnosis and the importance of resilience. In response to traumatic stress, some individuals may not report symptoms, some may develop psychological symptoms that resolve rapidly, while others may develop psychiatric problems such as post-traumatic stress syndrome.^8^ Factors such as personality traits, social support and coping style are reported to contribute to how WLWH adapt to HIV-related stress.^9^

It is important to note that variations exist regarding individuals’ ability to adapt to stress. While some individuals exposed to adversity are not able to adapt, a good number of others are able to adapt with no apparent disruptions in their ability to function or to relate to others.^10^ The concept of positive adaptation after exposure to considerable adversity is referred to as resilience. Resilience is the ability to function competently following exposure to stressors and/or to achieve positive relational, cognitive and behavioural outcomes and healthy psychological and physical functioning despite adverse experiences.^11^ Mullins et al.^12^ described resilience as a process by which an individual learns to overcome the negative effects of risk exposure (e.g. diagnosis of HIV), cope with traumatic events (e.g. invasive medical procedures) and avoid negative trajectories of adjustment outcomes (e.g. increase uncertainty, depression and post-traumatic stress). It reflects the ability of an individual to maintain a stable equilibrium despite adversity.^10^ Thus, the outcome of resilience process in response to adversity is positive adaptation, effective coping and/or mastery.^13^

Resilience had been considered as a measure of coping from exposure to stress^14^ and associated with better treatment outcomes as well as improved well-being among WLWH.^9,15^ It can therefore be an important target for intervention in managing stress among WLWH in Nigeria. Following a scoping review between April and July 2017, the authors of this study did not find any literature on the relationships between perceived psychological stress and psychological resilience among WLWH in Nigeria. Therefore, the purpose of this study was to assess the relationship between HIV-related perceived stress and psychological resilience among WLWH in Niger state, Nigeria.

The current study is supported by Lazarus and Folkman^16^ model of stress. The model proposed that stress involves interaction between individuals and their environment in which demands exceed their resources and threaten well-being. It affirms that individuals can draw on available resources to cope with stress. The individual may draw on personal characteristics such as psychological resilience (internal resources) and/or environmental assets such as social support (external resources). The theory highlights the significance of cognitive appraisal of individuals’ ability and personal resources to cope with stress. With the scarcity of literature on resilience among WLWH in SSA and Nigeria in particular, this study may add a significant finding to the body of knowledge about the relationships of stress and resilience among this vulnerable population.

### Hypothesis

There are no significant relationships between perceived stress, resilience and demographic characteristics of WLWH in Niger state.

## Methodology

This quantitative study focuses on the relationship between perceived HIV-related stress and psychological resilience among WLWH. This study is part of a larger study on resilience among WLWH in Niger state, Nigeria.

### Study design

This cross-sectional predictive correlational design was used to assess the relationship between psychological resilience and stress among WLWH in Niger state, Nigeria.

### Setting

This study was conducted in three selected hospitals in Niger state, Nigeria. Niger state is one of the 33 states in Nigeria. It is in the north central geopolitical region of the country. The state has 25 local government areas distributed across its three geo-political zones. The major languages spoken in the state are Nupe, Hausa and Gwari.

The hospitals where the data were collected were Umaru Sanda Ndayako General Hospital Bida, General Hospital Minna and General Kontagora. These hospitals are located each in zone A, zone B and zone C of the state, respectively. The three hospitals are public healthcare facilities that provide primary care including care for people living with HIV. Individuals from rural settings access HIV care in these selected hospitals.

### Population and sampling

The target population for this study was WLWH attending three selected hospitals in Niger state, Nigeria. A cluster sampling strategy was used to select the three hospitals across the three geopolitical zones of Niger state in northern Nigeria.

In consultation with the school statistician, the following parameters were used to determine the sample size:

alpha error (type 1) = 0.05

beta error (type 2) = 0.2

statistical power = 1 − 0.2 = 0.8

number of predictors = 5: resilience, age, marital status, level of education and employment status.

Based on the above parameters, a sample size of 748 was determined. A systematic random sampling technique was used to recruit 249, 249 and 250 participants from the 2650, 2472 and 2710 women enrolled in HIV clinics of General Hospital Bida, General Hospital Kontagora and General Hospital Minna, respectively. The inclusion criteria were as follows: the participant must be a woman, diagnosed with HIV for not less than 4 months, have been 18 years old and above, and have been enrolled on ART for not less than 3 months.

### Procedure

Data were collected between 25 September and 24 December 2017. The data were collected using a self-report questionnaire with validated and reliable scales. Each participant was administered the perceived stress scale (PSS) and Connor–Davidson resilience scale (CD-RISC) questionnaires for completion. The English version of the instrument was translated and back-translated into Nupe and Hausa languages to accommodate participants who do not understand English language. The translations were done by two independent language experts in Niger State College of Education Minna.

### Pilot study

After translation, the instrument was pilot-tested among 10 participants who were WLWH for each language. Comments and observations were noted, and minor corrections were made. The reliability of Cronbach’s alpha obtained for the scales was > 0.7 and > 0.8 for resilience and PSS, respectively.

### Data collection

Three research assistants administered the questionnaire in each of the selected hospitals. After informed consent, individuals who agreed to participate were handed the questionnaire to complete and return on the spot. Participants who could not read were assisted by the research assistants in offices provided within the clinics. Other information elicited in the questionnaire include age, marital status, employment and number of children.

From a total of 748 questionnaires distributed to WLWH across the three selected hospitals, 676 returned the questionnaires in usable forms, giving a return rate of 90.1%. The returned questionnaires were 224, 221 and 231 from General Hospital Bida, General Hospital Kontagora and General Hospital Minna, respectively. Only 17, 23 and 21 individuals declined participation in the study from General hospitals Bida, Kontagora and Minna, respectively.

### Measures

#### HIV-related stress

Perceived stress is described as the extent to which a situation in one’s life during the past 1 month was perceived as stressful.^17^ The PSS^17^ was used to measure HIV-related stress in this study. The instrument is reliable and correlates with life-event scores and anxiety.^17^ The scale consists of 14 items that elicit information from the respondents about their exposure to stressful events. Participants indicated the frequency with which they felt stressed over the past 1 month on a five-point Likert scale comprising the following scores: never (0), almost never (1), sometimes (2), fairly often (3) and very often (4).

To obtain the scores in the PSS, the seven positively worded items were reversed and then summed across all the 14 items, with greater scores indicating high level of perceived stress. The scores that are obtainable range from 0 to 56. The scale is reported to have good validity and reliability. The reliability alpha coefficient for PSS is 0.85 and the test–retest correlation was 0.85.^17^

#### Resilience measure

The CD-RISC^14^ was used as a measure for resilience. It has 25 items rated on a five-point Likert scale of 0–4. Scores obtained by each participant in all the 25 items are summed. The obtainable score ranges from 0 to 100. Higher scores denote higher perceived resilience. The Cronbach’s alpha of the scale was > 0.7. The scale was developed using multiple samples in different settings, including clinical settings and general populations. However, most of the samples were consisted of women.^14^

### Data analysis

The data analysis was performed using Statistical Package for the Social Sciences (version 25). Descriptive statistics were used to describe the demographic characteristics of the participants, level of perceived stress and level of perceived resilience. Pearson’s correlation coefficients and standard linear regression analysis were used to examine the relationships between perceived stress (outcome), resilience and demographic variables (age, marital status, level of education and employment status).

### Ethical consideration

Ethical approval to conduct the study was obtained from the Biomedical Research Ethics Committee of the University of Kwazulu-Natal, South Africa, with protocol reference number: BE296/17. Niger state Ministry of Health and ethics committee of General hospitals Bida, Minna and Kontagora also gave approval for the study. Informed consent was obtained from each participant and the participants were assured that the data will only be used for research purpose prior to questionnaire administration. They were also informed that participation is voluntary.

## Results

### Demographic characteristics of respondents

The results in Table 1 show the demographic distribution of the respondents. The mean age of the respondents in the sample was 36.90 ± 12.2 years and they were predominantly Muslims and Christians in terms of religion. The major ethnic groups of the respondents were Hausa, Nupe and Gwari. Other different minor ethnic groups made up 35.5% of the respondents. The majority (71.4%) of the respondents were married, while those that were single, divorced and widowed made up of 14.2%, 7.4% and 7.0% of the respondents, respectively. Most (70.3%) of the respondents had 1–4 children; 11.2% of them had 5–8 children, while 18.5% of them had none. Data on their highest level of education showed that most (34.3%) of the respondents had secondary education, while those that had primary, diploma and tertiary educations were 19.1%, 12.7% and 3.3%, respectively. However, many (30.6%) of them had no formal education. The employment status of the respondents showed that the majority (67.9%) of them were unemployed, while only 32.1% were employed.

**TABLE 1:** Demographic distribution of the respondents (*N* = 676).

Demographic characteristics	No. of respondents	$\bar{\text{X}}$/%
Age in years	676	36.90± 2.2
**Religion**		
Islam	337	49.9
Christianity	325	48.1
Others	14	2.1
**Ethnicity**		
Hausa	171	25.3
Nupe	134	19.8
Gwari	131	19.4
Others	240	35.5
**Marital status**		
Married	483	71.4
Single	96	14.2
Divorced	50	7.4
Widowed	47	7.0
**No of children**		
0	125	18.5
1–4	475	70.3
5–8	76	11.2
**Level of education**		
No formal education	207	30.6
Primary	129	19.1
Secondary	232	34.3
Diploma	86	12.7
Tertiary education	22	3.3
**Employment status**		
Employed	217	32.1
Unemployed	459	67.9

Results on perceived stress among the participants revealed a mean score of 26.93 ± 6.35. The highest perceived stress levels were perceived in the respondents’ ‘inability to control the important things in their life’ and ‘inability to control irritations in their life’, with cumulative scores of 2153 and 2072, respectively. Life events the participants least perceived stresses were when they ‘dealt with irritating life hassles’ and when they ‘felt that things are going their way’, with a cumulative score of 1501 and 1670, respectively.

The mean score of the resilience measure in this study was 60.04 ± 12.47. The highest resilience scores were recorded in the participants’ perception that ‘past success gives confidence to new challenge’ (2487), ‘making unpopular or difficult decisions’ (2416) and ‘putting in best effort no matter what’ (2408). Furthermore, the lowest resilience scores were observed in their perceived ability to ‘focus and think clearly under pressure’ (1965), ‘seeing humorous side of things’ (2061) and ‘coping with stress strengthens’.

The results in Table 2 show that the mean scores of perceived stress and resilience among the sample across the three selected hospitals were not statistically different.

**TABLE 2:** Comparison of mean scores of resilience and perceived stress across the selected hospitals.

Setting	Perceived stress $\bar{\text{X}}$ and s.d.	Resilience $\bar{\text{X}}$ and s.d.	*n*
Bida	27.50 ± 7.28	58.93 ± 13.56	224
Kontagora	26.87 ± 6.71	61.06 ± 10.92	221
Minna	26.46 ± 4.81	60.14 ± 12.67	231

s.d., standard deviation; $\bar{\text{X}}$, mean.

The relationships between resilience, perceived stress and marital status were assessed using Pearson’s product–moment correlation after preliminary analysis to ensure that there were no violations of assumptions. The results in Table 3 show a significant negative relationship between HIV-related stress and psychological resilience among WLWH in Nigeria (*p* < 0.05). This implies that high level of psychological resilience is associated with lower perceived stress. The findings also revealed no significant relationships between age and resilience, age and perceived stress, marital status and resilience, and marital status and perceived stress (*p* < 0.05).

**TABLE 3:** Relationship between perceived stress and resilience among women living with HIV in Nigeria (*N* = 676).

Variables	Pearson *r*	*p*
Resilience and perceived stress	−0.601	< 0.001
Age and resilience	0.063	0.100
Age and perceived stress	−0.044	0.249
Marital status and resilience	0.051	0.189
Marital status and perceive stress	−0.001	0.973

A standard linear regression analysis was performed to test the ability of resilience measure and demographic variables (age, marital status, level of education and employment status) of the respondents to predict the level of perceived stress (PSS). The results in Table 4 show that 36.3% of the variance in perceived stress can be explained by psychological resilience and demographic variables of the respondents. The results also show that only the resilience measure was statistically significant among the independent variables (*p* < 0.001). This implies that higher level of psychological resilience is significantly associated with lower level of perceived stress. Age, marital status and education were not significant predictors of perceived stress in the analysis.

**TABLE 4:** Results of multiple linear regression (*N =* 676).

Independent variables	Coefficients	s.d.	*p*	Confidence interval
Resilience	−0.305	0.022	< 0.001	−0.347 to -0.263
Age	−0.033	0.016	0.794	−0.036 to 027
Marital status	0.267	0.237	0.261	−0.199 to 733
Education	0.81	0.173	0.640	−0.258 to 420
Employment	−0.300	0.575	0.603	−1.43 to 0.830

s.d., standard error; constant 45.2; *p* < 0.05.

## Dependent variable: Perceived stress

### Discussion

Although the relationships between stress and psychological resilience have been established among people living with HIV,^18^ little is known about this relationship among WLWH in SSA in general and Nigeria in particular. Following a scoping review between April and July 2017, there were no publications on the association between resilience and stress among WLWH in Nigeria. To the best of our knowledge, this is the first study that explored the relationships between HIV-related stress and psychological resilience among WLWH in Nigeria.

In the current study, the mean perceived HIV-related stress was 26.93, indicating a moderate perceived stress. This finding is inconsistent with what was reported in another study^19^ in which the mean (*M* = 9.3) perceived HIV-related stress was low among the study population. Coping with multidimensional stressors among WLWH is a difficult task particularly in SSA, the epicentre of the disease. A qualitative study on psychosocial stressors of HIV-infected women identified difficulties with status disclosure, ART adherence, stigma and discrimination, relationship challenges, financial difficulties and parental roles as common stressors.^20^ Other evidence indicates that PLWHA, especially women, are still exposed to high levels of psychological distress, especially through stigma and discrimination.^21,22,23,24,25,26,27,28^ In Nigeria, a study revealed that psychosocial stress among WLWH may be related to socio-economic status, poor family and support system, stigma and discrimination.^29^ Moreover, stress is documented to be associated with poor quality of life and severe physical symptoms of HIV,^30^ disease progression, low CD4 count, development of AIDS and increased risk of death among PLWH.^31^

Again, the level of stress among the participants may place them at higher risks of mental disorders as it is in both general population and settings with social inequalities.^32^ Although this might differ from one context to another depending on economic, health and social infrastructure available,^32^ several evidences had shown that, among the factors, stigma had been identified as a key player associated with severe physical and psychological stress, severe symptoms and lower CD4 counts among people living with HIV compared with people with other medical conditions.^30,33,34,35,36,37,38^

The results also show that most of the respondents were unemployed and most did not attend primary education and educational status not significantly associated with perceived stress. Contrary to the findings of this study, evidence had shown that education and employment status were found to ameliorate stress and improve resilience among WLWH. Those with higher education and employment are more resilient than those who are not.^39^ Apart from the financial benefits of employment, individuals who are employed may learn new skills from colleagues and engage in activities that reduce lethargy, boredom, stress and depression.^39^ Individuals who are employed are more mentally fit and may avoid negative behaviours such as use of alcohol and drugs to cope with stress.^40^ Similarly, people with higher education are more likely to have better psychological well-being and cope with life challenges such as HIV.^41^

The findings of this study show that the sample had high-level resilience, with a mean score of 79.1. This finding is consistent with a study conducted in Chicago, USA,^9^ that reported high level of resilience among WLWH. This finding is also consistent with the study by Fang et al.^18^ who found resilience to be associated with life stress and psychological and physical well-being of people living with HIV. Contrary to the finding of this study, Spies and Seedat^42^ in a study on the relationship between depression and resilience among women with HIV found a significant negative relationship, implying that resilience buffered the effect of life stress. It is pertinent to state that resilience promotion is essential to ameliorate the effects of HIV-related stress among WLWH. However, an unexpected result from this study is that resilience and stress were not significantly correlated with age and marital status of the participants.

### Limitation

Firstly, the finding of this study cannot be used to establish causality because of the use of cross-sectional design. Secondly, the use of selected hospitals and limited sample size limits the generalisability of the study results.

## Conclusion and recommendation

This study highlights the association HIV-related stress and psychological resilience among WLWH in Nigeria. It is therefore important to integrate resilience training into mental health services in SSA where women are disproportionately affected by psychological stress related to HIV. We recommend the need to explore the impact of resilience demographic variables of WLWH in a longitudinal study.
